# Expression of Nucleolin Affects Microtubule Dynamics

**DOI:** 10.1371/journal.pone.0157534

**Published:** 2016-06-16

**Authors:** Xavier Gaume, Christophe Place, Helene Delage, Fabien Mongelard, Karine Monier, Philippe Bouvet

**Affiliations:** 1 Université de Lyon, Ecole normale Supérieure de Lyon, Centre de Recherche en Cancérologie de Lyon, Cancer Cell Plasticity Department, UMR INSERM 1052 CNRS 5286, Centre Léon Bérard, Lyon, France; 2 Université de Lyon, Ecole Normale Supérieure de Lyon, Université Claude Bernard, CNRS, Laboratoire de Physique, F-69342, Lyon, France; University of Illinois at Chicago, UNITED STATES

## Abstract

Nucleolin is present in diverse cellular compartments and is involved in a variety of cellular processes from nucleolar structure and function to intracellular trafficking, cell adhesion and migration. Recently, nucleolin has been localized at the mature centriole where it is involved in microtubule nucleation and anchoring. Although this new function of nucleolin linked to microtubule regulation has been identified, the global effects of nucleolin on microtubule dynamics have not been addressed yet. In the present study, we analyzed the roles of nucleolin protein levels on global microtubule dynamics by tracking the EB3 microtubule plus end binding protein in live cells. We have found that during microtubule growth phases, nucleolin affects both the speed and life time of polymerization and by analyzing catastrophe events, we showed that nucleolin reduces catastrophe frequency. This new property of nucleolin was then confirmed in a cold induced microtubule depolymerization experiment in which we have found that cold resistant microtubules were totally destabilized in nucleolin depleted cells. Altogether, our data demonstrate a new function of nucleolin on microtubule stabilization, thus bringing novel insights into understanding the multifunctional properties of nucleolin in healthy and cancer cells.

## Introduction

Microtubules are highly dynamic constituents of cell cytoskeleton. They are tubular polymers of the α-β tubulin heterodimers emanating from nucleating centers like the centrosome. They are important during interphase to maintain cell shape and polarity, to organize intracellular trafficking to control organelle positioning, and to favor cell motility during migration. In mitosis, microtubules are drastically reorganized and their dynamics considerably increased. Indeed, by forming the bipolar mitotic spindle, microtubules are key actors for chromosome segregation between daughter cells.

Microtubules constantly switch between growth phases (polymerization) and catastrophes (fast depolymerization). This behavior known as dynamic instability [1] is an important feature to allow microtubule functions. A precise regulation of microtubule dynamics is essential for controlling major cellular processes in a healthy cell. As microtubules play important roles in cancer related cellular processes they are key targets for therapeutic treatments against cancer and other pathologies [2]. Indeed, several human tumor suppressor genes and oncogenes have microtubule regulating functions: APC [3], Axin [4], NF2 [5], B23 [6] and pVHL [7]. Thus, understanding how microtubules are regulated in normal cells could identify new targets to prevent cell proliferation and invasion in a tumor environment.

Nucleolin (NCL) is one of the most abundant non ribosomal proteins of the nucleolus where it plays a central role in ribosome biogenesis. Nevertheless, its functions are not restricted to nucleoli. Indeed, several functions of nucleolin have been described in the nucleoplasm, cytoplasm and at the cell membrane. Through its numerous sub cellular localizations and functions, nucleolin has been involved in the initiation and progression of lots of cancers [8] and more specifically in processes that could involve microtubules dynamics like cell adhesion and migration [9–12] as well as intracellular trafficking [13, 14].

We have recently identified a new function of nucleolin linked to microtubule regulation [15]. Indeed, nucleolin has been localized to the mature centriole of interphase cells. Through its interaction with ninein and γ-tubulin, nucleolin controls microtubule nucleation and anchoring at the centrosome [15]. Nucleolin depletion leads to microtubule disorganization in interphase cells [15]. Interestingly, these effects are similar to that of another nucleolar protein: B23 [15–17]. Thus, although nucleolin and B23 have different structural domains, they share similar functions, not only in the nucleoli, but also related to the centrosome and microtubules. Recently, B23 has been shown to stabilize microtubules in vitro by inhibiting the kinesin Eg5 [6]. Although the association of nucleolin with microtubules has been identified in a proteomic study, (supplemental Table S1 in [18]), the interaction and the effects of nucleolin on microtubule dynamics have not been addressed.

Here we have addressed the consequences of nucleolin expression (depletion by siRNA or over expression of exogenous protein) on parameters of microtubule dynamics by analyzing EB3 kinetics, a microtubule plus end binding protein. For this analysis, we used a Matlab-based open source software package to track EB3 and measure microtubule dynamics. The effects obtained on microtubule dynamics in cells under- or over-expressing nucleolin were compared to the ones obtained in cells under- or over-expressing B23. We found that during growth phases, nucleolin controls growth speed and favors polymerization by decreasing the frequency of pauses and catastrophe events. In addition, absence of nucleolin was found to increase the amplitude of depolymerization (speed and length). In agreement with that, microtubule resistant to a cold treatment were lost in cells depleted in nucleolin. Altogether, our results reveal an important new role for nucleolin in microtubule dynamics.

## Materials and Methods

### Antibodies and Reagents

Nucleolin was detected with a rabbit polyclonal antibody raised against purified human nucleolin (pab0971-P Covalabs / dil. WB 1/1000) as previously described [15, 19]. B23 was detected with a mouse monoclonal antibody (FC82291 Sigma-Aldrich #B0556 / dil. WB 1/1000). α-tubulin was detected with a mouse monoclonal antibody (DM1A Sigma-Aldrich #T9026 / dil. WB 1/1000). β-actin was detected with a mouse monoclonal antibody (AC-15 Sigma-Aldrich #A5441 / dil. WB 1/1000). Acetylated tubulin was detected with a mouse monoclonal antibody (611B1 Sigma-Aldrich #T7451 / dil. IF 1/300). GFP detection was boosted with an anti GFP antibody directly coupled to Atto488 (Chromotek / dil. IF 1/200). For Immunofluorescence, secondary antibodies were coupled to Alexa555 (Molecular Probes DaMAlexa555 #A31570 / dil. 1/2000). For WB, secondary antibodies were coupled to IRdye800 (Li-Cor #92632211 / dil. 1/2500) or Alexa680 (Li-Cor #92632220 / dil. 1/15000). EB3-tagRFP plasmid has been previously described [15].

### Cell culture and transfection

U2OS-centrin-1-GFP cells (a kind gift from M. Bornens) [20] stably expressing EB3-tagRFP were obtained as described in [15] and were grown in Dulbecco’s Modified Eagle Medium DMEM (PAA #E15-883) supplemented with 10% fetal calf serum (FCS) (PAA #A15-151), 1% of non essential amino acids (PAA #M11-003), 1% of penicillin-streptomycin (PAA #P11-010) and 1mM of Sodium pyruvate (PAA #S11-003). Accutase (GE Healthcare #L11-007) was used for cell detachment.

For nucleolin siRNA, a mixture of small interfering RNA (siRNA) specific for human nucleolin was used as previously described [15, 19, 21, 22]. For B23 silencing, a mixture of siRNAs specific for human B23 was used (Invitrogen B23-HSS143152 GAUGGAACUCCACCCUUUGCUUGGU and B23-HSS143153 UGUAUGGAAUGUUAUGAUAGGACAU) [23]. All siRNAs were reconstituted at a concentration of 100μM and stored at -20°C. As a control siRNA, we used Stealth high GC siRNA (Invitrogen).

Cells were transfected in 6-well dishes using siRNA at 20nM final concentration. siRNAs were diluted in 200μl of OptiMEM and plated in a well. 80μl of INTERFERin (Polyplus) diluted 1/10 in RNase-free water were added. After 10 min incubation, 2ml of medium containing 3.10^5^ cells were added. After 2 days, cells were detached and plated in 24-well dishes onto glass coverslips for further immunofluorescence or plated in 10cm dishes for western blot analysis or in 35mm dishes for live cell imaging (μ-dish glass bottom Ibidi treat Biovalley #81158). Cell fixation, lysis and imaging were performed 96h after siRNA transfection. For over expression experiments, U2OS-C1G/EB3-tagRFP cells were transfected with B23-GFP [pEGFP-hB23-C1 a kind gift of T. Pederson [24]] or NCL-GFP [NCL-19aaGFP: NCL and GFP sequences are spaced out by 19 amino acids (Delage and Bouvet unpublished)].

For plasmid transfection, one day before transfection, 25000 cells were plated in 35mm dishes for live cell imaging (μ-dish glass bottom Ibidi treat Biovalley #81158). Cells were transfected in CM-E medium using jetPRIME DNA transfection reagent (Polyplus transfection) as recommended by the manufacturer (1μg of DNA and 2μl of jetPRIME reagent).

### Western blot analysis

Cells were detached, lysed in 2% SDS, 10% glycerol and 20% β-mercapto-ethanol for a final concentration of 1.10^4^ cells/μl, and heated at 95°C for 5min. Lysate from 1.10^5^ cells was loaded onto a 10% SDS polyacrylamide gel electrophoresis. The proteins were then transferred to Protran membranes (Protran BA85, Whatman, /GE Healthcare/ #/10 401 196). Membranes were blocked in 5% milk and incubated with the primary antibodies over night at 4°C in PBS containing 1% milk, then washed in PBS and secondary antibody incubations were performed for 30min in PBS containing 1% milk at room temperature. Monoclonal antibodies were detected with a secondary antibody coupled to Alexa680, while polyclonal antibodies were detected with a secondary antibody coupled to IRdye800. After PBS washes, western blot imaging were acquired with an Odyssey infrared system (LiCor Biosciences).

### Immunofluorescence

Cells were plated 5.10^4^ cells/well in 24-well dishes onto glass coverslips. 2 days after plating, cells were fixed in cold methanol for 3min at -20°C and then permeabilized with 0.1% Triton X-100 in PBS (PBS-T) for 2 x 10min. All subsequent incubations were performed in a humidified chamber at 37°C. Non specific binding of antibodies was blocked by 10% FCS, 3% BSA and 0.1% Triton X-100 in PBS (blocking buffer) for 30 min. Coverslips were next incubated with primary antibodies diluted in the blocking buffer for 30min. After 3 washes at room temperature in PBS-T, they were incubated with secondary antibodies also diluted in the blocking buffer, for 30min. After 3 more washes in PBS-T, coverslips were washed in PBS, rinsed in ddH_2_O and briefly dipped in absolute ethanol. After a quick dry, coverslips were mounted on a slide with Fluoromount G (FMG Southern biotech *#0100–01*) containing 400ng/ml 4',6'-diamidino-2-phenylindole (DAPI).

### Microscopic image acquisition and treatment

Twelve-bit images were acquired using a Cool Snap HQ charge-coupled-device (CCD) camera mounted on a Zeiss Axio-Imager Z1 equipped with a 63x oil-immersion objective lens (numerical aperture = 1.4 / working distance 0.19mm) and flImager Z1 filters suited for the visualization of DAPI, Atto488 and Alexa555. For each field of view, z-stacks of about 25 images with a pixel size of 102 nm were obtained by setting the z-step at 200 nm. Images stacks were processed using a 3D constrained iterative deconvolution module running under Metamorph (Meinel Algorithm on Metamorph [iteration: 7x / σ: 0.7 / frequency: 5 / without auto background]), using the Point Spread Functions (PSF) measured for the different channels under similar acquisition conditions for PS-speck beads (Molecular probes) mounted in the same mounting medium. For each analyzed cell, the optical section in which the centrosome was the most in focus was chosen. The x and y shifts between individual channels were corrected on the 3D stack by imaging 100nm multi fluorescent microspheres under similar acquisition conditions (translation of red channel x-1).

For the time-lapse acquisition of EB3-tagRFP signal, U2OS-centrin-1-GFP cells stably expressing EB3-tagRFP were imaged by TIRF microscopy 96 hours after siRNA transfection. Images were acquired using an EMCCD camera (Hamamatsu C9100, pixel size: 0.16μm) mounted on a TIRF microscope (Leica DMI6000) equipped with a 100x oil-immersion objective (Leica / numerical aperture = 1.46). Images were acquired every 0.8s during 3min at 37°C in a thermo regulated atmosphere (TIRF penetration 150nm / laser line 561nm / Quad filter 405-488-561-635nm).

Signal was then tracked in each individual cell using Matlab (MathWorks R2012b) and the PlusTipTracker open source software package [25, 26] with the following parameters: σ1 = 1 / σ2 = 4 / K = 3 / Search radius range = 1–5 pixels / Minimum sub-track length = 3 frames / Maximum gap length = 30 frames / Maximum shrinkage factor = 1.5 / Maximum angle forward = 30° / Maximum angle backward = 10° / Fluctuation radius = 2 pixels. Results tables were then exported and further analyzed using Excel (Microsoft Office). Growth, pause and catastrophe events were analyzed by selecting all the type 1, 2 or 3 subtracks respectively. EB3-TagRFP movies were acquired in three independent experiments.

### Statistical analysis

For the statistical analysis, the mean value of each dynamic parameter (speed / life time / displacement / pause frequency / catastrophe frequency) was individually calculated in each entire cell area. Data were then directly reported on the 2D graphs. Then, for each condition, the mean value of the dynamic parameter was obtained by averaging the value calculated for each cell. On the graphs, boxes indicate 25 and 75% quartiles and the whiskers extend to 1.5 times of the interquartile range. Outlier cells are not shown. Statistical significance between the different conditions was assessed using the nonparametric Wilcoxon-Mann-Whitney two-sample rank test [27]. This test can be applied on unknown distributions contrary to *t*-test which has to be applied only on normal distributions. The Wilcoxon-Mann-Whitney test combines two samples and ranks them to test the hypothesis that the data in the two input samples are statistically the same. The distribution is computed using Igor software (Wavemetrics). We discriminate significant levels at 0.1, 0.05 and 0.005.

### Determination of the Normalized microtubule density and of the cell shape

Among a z-stack of 26 images, the best focus image was selected, Fast Fourier Transformed and a high-pass filter was applied in both x and y to enhance the microtubule width detection. Inverse Fast Fourier Transform restituted the cleaned original image and an automatic thresholding method [28] was applied for binarization. Using a SeedFill algorithm. The whole cell surfaces areas were determined and used to calculate the normalized microtubule density: microtubule surface divided by the whole cell surface. Building of kymographs: A single microtubule comet that appeared for more than 30 s in a time series image stack (image rate: 800ms) was selected for each condition. The image around the comet was reduced to a one pixel (160 nm) slice and for all images in the stack, the slices were concatenated in perpendicular direction to produce the kymograph. All image manipulations and computations are performed with the software Igor pro 6.36 (WaveMetrics).

For the cell shape analysis, single cell or cell agglomerates were analysed using the AnalyzeParticles operation (Igor software) to determine their surface area, circularity and perimeter. Circularity is define as the ratio of the square of the perimeter to (4*π*Area). Numbers of cell were determined by counting the DAPI labelled nuclei.

## Results

### Nucleolin expression level affects the dynamics of growing microtubules

**Fig 1:**
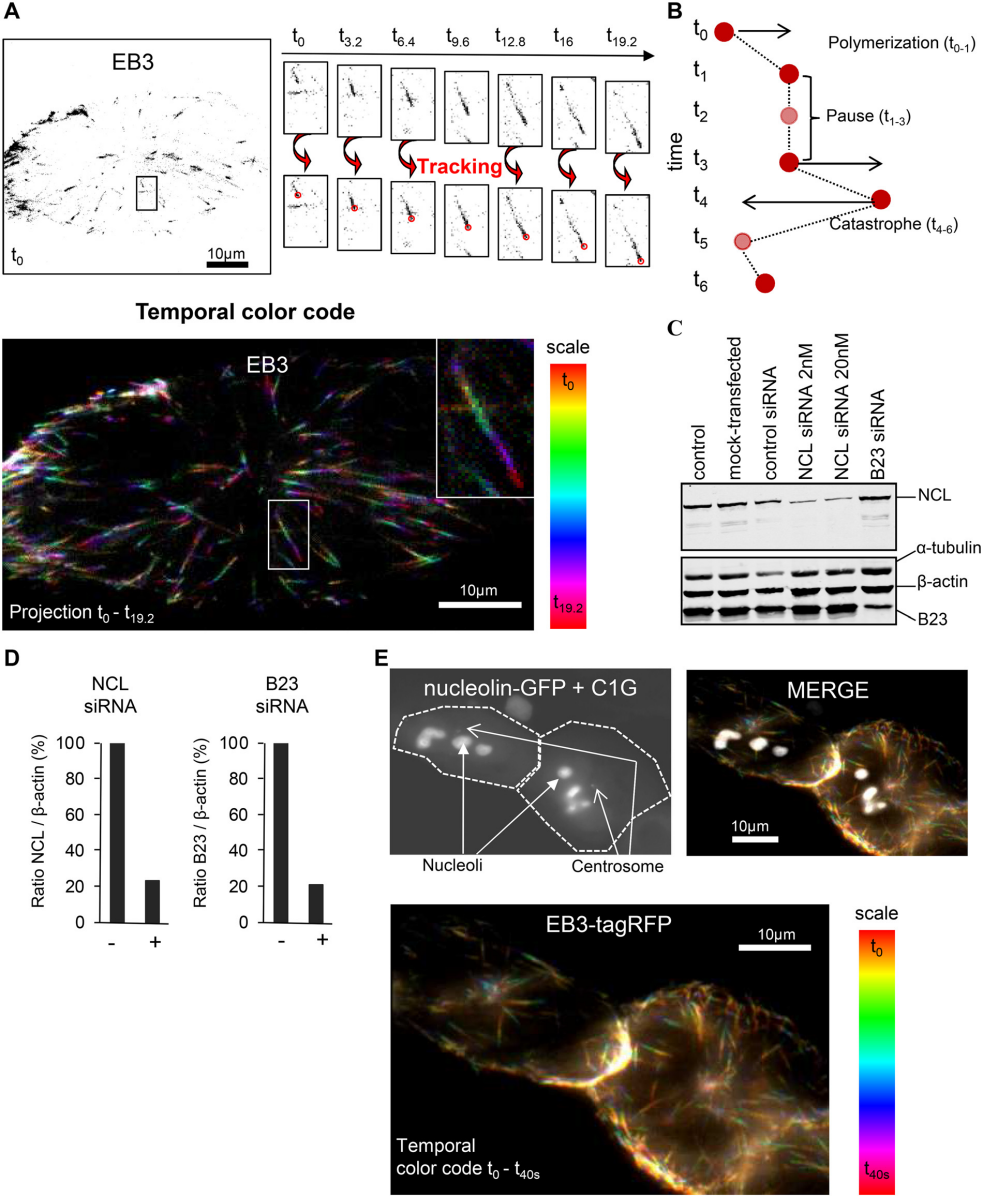
Microtubule dynamics analysis using the EB3-tagRFP plus-end tracking protein. (A) Still frames from time-lapse experiments show EB3-tagRFP signals. Upper left image represents EB3-tagRFP signal at t = 0s. On the right of the image, enlarged images of an EB3-tagRFP comet evolution (displayed on the full size image) are shown (upper enlarged panels from t = 0s to t = 19.2s). The plusTipTracker software package detects and tracks all the microtubule growing ends as exemplified on the enlarged images (lower enlarged panels). Lower image represents a temporal color code image of the EB3-tagRFP signal evolution from t = 0s to t = 19.2s. The color code scale is presented on the right. Scale bars represent 10μm. (B) Schematic representation of EB3-tagRFP tracking using the plusTipTracker software. Microtubule growing ends are schematized with the red circles. Transparent red circles represent temporarily comet disappearance. After comet detection and tracking, the software classifies the sub-tracks in three groups: polymerization, pause and catastrophe. For more details see [25][27–30]. (C) Inhibition of nucleolin or B23 expression by siRNA. Western-blot analysis of whole cell extracts from untransfected control, mock transfected, control siRNA, nucleolin siRNA (siRNA concentration 2nM or 20nM) and B23 siRNA transfected cells. Cells were harvested four days after transfection and protein extracts were analyzed by western-blot using a nucleolin polyclonal antibody (detected with a secondary antibody coupled to IRdye800) and B23, α-tubulin and β-actin monoclonal antibodies (detected with secondary antibody coupled to Alexa680). (D) Quantifications of the fluorescent western blots, displayed in (C), expressed as a ratio of nucleolin over β-actin (left) or B23 over β-actin (right). The normalized expression level of nucleolin or B23 proteins was set to 100% in control cells (NCL siRNA 22.60% and B23 siRNA 20.59%). (E) Nucleolin over expression by transient transfection of a NCL-GFP construct. On the upper left image, the GFP channel is presented [black and white]. Arrows show nucleoli (nucleolin-GFP signal) and the centrosomes (C1G: Centrin-1-GFP signal) and cell outlines are shown. Lower image represents a temporal color code image of the EB3-tagRFP signal evolution for 40s. The color code scale is presented on the right of the image. Upper right image merge image of the GFP channel and the temporal color code image of the EB3-tagRFP signal is presented. Scale bars represent 10μm.

To further investigate the functions of nucleolin and B23 on microtubule regulation, we have studied the effects of changes in their expression levels (silenced, normal and over-expressed) on microtubule dynamics by analyzing the dynamics of the EB3 plus-end tracking protein. EB3 is a useful marker to analyze microtubule polymerization (Fig 1A and 1B) [27, 28]. We used a U2OS osteosarcoma cell line stably expressing the EB3-tagRFP and centrin-1-GFP proteins (previously described in [15]. Transfection with siRNAs was used to efficiently decrease nucleolin and B23 protein levels (Fig 1C and 1D) whereas over expression of these proteins was obtained by transient transfection of a NCL-GFP (Fig 1E) or a B23-GFP expressing construct.

For high-throughput measurement, we analyzed the dynamics of EB3-tagRFP signal using PlusTipTracker, a Matlab-based open source software package, which automatically detects and tracks thousands of EB3-tagRFP comets on the movies [25, 26] and provides microtubule characteristics during growth phases (Fig 1B, see polymerization). We were also able to deduce microtubule characteristics during pauses and catastrophe events, in which the EB3-tagRFP comets temporarily disappear (Fig 1B, see pause and catastrophe) [29]. Altogether, this assay allows the analysis of the effects of changing nucleolin and B23 protein levels on global microtubule dynamics in live cells.

**Fig 2:**
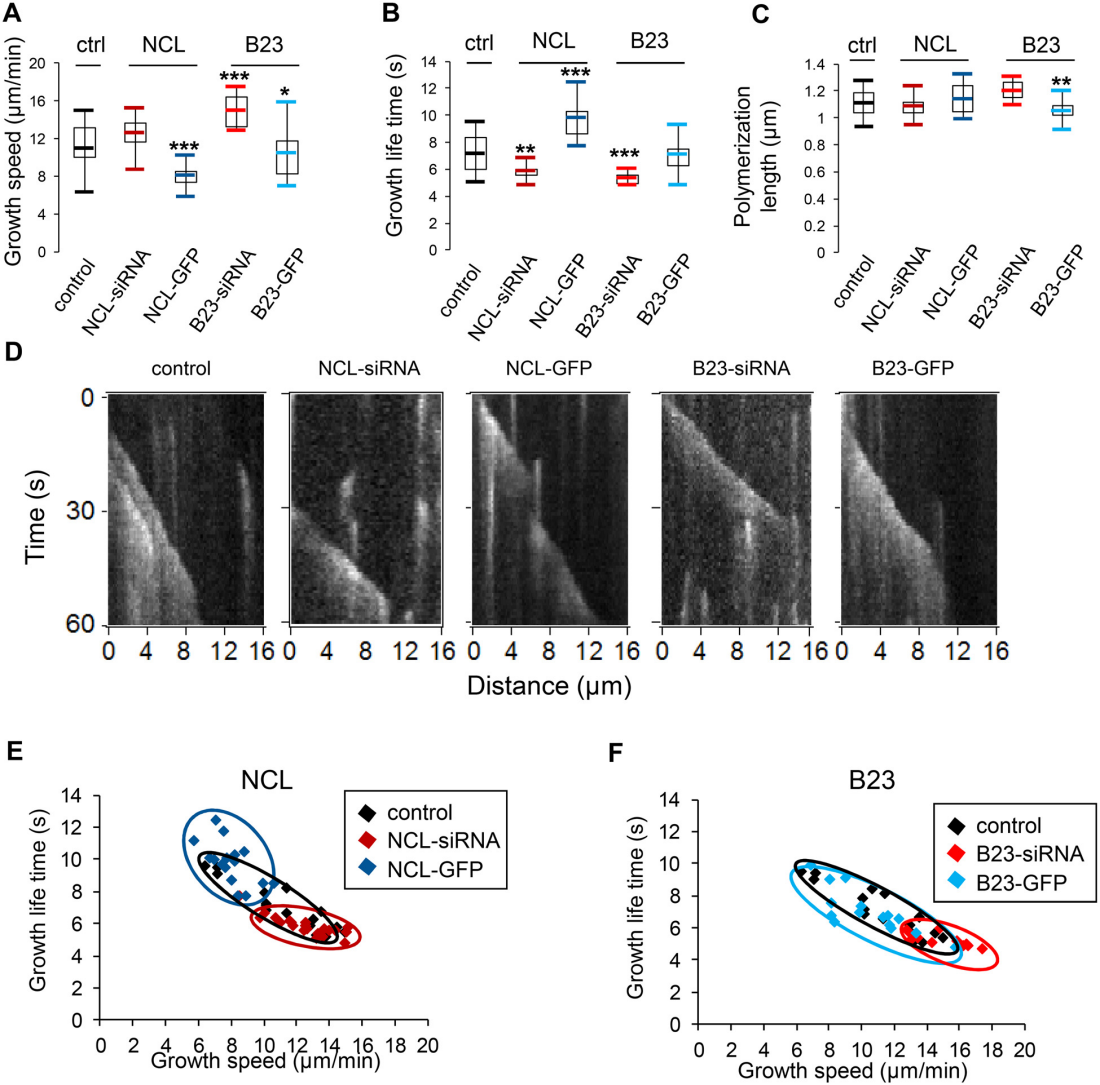
Nucleolin expression affects microtubule growth. (A)-(C): Box and whisker plots represent tracks for growth speed (A), growth life time (B) and growth displacement (C). For each condition (control [black], nucleolin depletion [dark red], nucleolin over expression [dark blue], B23 depletion [light red] and B23 over expression [light blue]), cells were individually analyzed (control: 18 cells; NCL-siRNA: 24 cells; NCL-GFP: 16 cells; B23-siRNA: 11 cells; B23-GFP: 15 cells). The mean value of the parameters was individually calculated in each cell. For each condition, the mean value of the parameters was obtained by averaging the value calculated for each cell. On the graphs, boxes indicate 25 and 75% quartiles and the whiskers extend to 1.5 times of the interquartile range. Outlier cells are not shown. The asterisks indicate that the mean values differ from the control condition with the Wilcoxon-Mann-Whitney two-sample rank test at different significance levels: 10% (* P<0.1), 5% (** P<0.05), 0,5% (*** P<0.005). (D) Kymographs of a single microtubule segment from the time-lapse series for each condition. (E)-(F) 2D graphs showing the repartition of the cells according to growth speed (x-axis) and growth life time (y-axis). Control [black], nucleolin over expressing [dark blue] and nucleolin depleted [dark red] or B23 over expressing [light blue] and B23 depleted [light red]. Cells are represented by the squares. Entire cell area were individually analyzed for each condition [control: 18 cells / NCL-siRNA: 24 cells / NCL-GFP: 16 cells / B23-siRNA: 11 cells / B23-GFP: 15 cells]. In all, thousands of microtubules were analyzed for each condition [control: 16262 microtubules / NCL-siRNA: 42737 microtubules / NCL-GFP: 10215 microtubules / B23-siRNA: 15915 microtubules / B23-GFP: 12664 microtubules]. The mean values of growth parameters are shown [speed (μm/min) / life time (s) / displacement (μm)]. σ is the standard deviation.

We first analyzed the effects of nucleolin and B23 protein levels on microtubule dynamics during polymerization phases (Fig 2; Table 1). Growth speed, growth life time and polymerization length were measured and averaged in individual cells. Different conditions were compared: control [ctrl] n = 18 cells / nucleolin depletion [NCL-siRNA] n = 24 cells / nucleolin over expression [NCL-GFP] n = 16 cells / B23 depletion [B23-siRNA] n = 11 cells / B23 over expression [B23-GFP] n = 15 cells). The results correspond to the mean values obtained in each condition (see Table 1). After nucleolin depletion we observed a slight increase in microtubule polymerization speed, which was not significantly different from the control (Fig 2A). However, in B23 silenced cells a significant growth speed increase was noticed compared to control cells when a Wilcoxon Mann Withney test was applied (P< 0.005) (Fig 2A). On the contrary, Nucleolin and B23 over expression had an opposite effect, decreasing significantly microtubule growth speed compared to control cells (P<0.005 and P<0.1 respectively) (Fig 2A).

**Table 1:** Microtubule growth parameters.

	Population		Growth		
	Cells (n)	Microtubules (n)	speed (μm/min)	life time (s)	displacement (μm)
control	18	16262	10.97 (σ = 2.70)	7.20 (σ = 1.49)	1.11 (σ = 0.11)
NCL siRNA	24	42737	12.53 (σ = 1.71)	5.78 (σ = 0.59)	1.09 (σ = 0.07)
NCL-GFP	16	10215	7.98 (σ = 1.21)	9.74 (σ = 1.33)	1.14 (σ = 0.12)
B23 siRNA	11	15915	14.91 (σ = 1.66)	5.29 (σ = 0.41)	1.20 (σ = 0.07)
B23-GFP	15	12664	10.42 (σ = 2.41)	7.07(σ = 1.38)	1.04 (σ = 0.10)

The number of cells (n) and microtubules (MT) (n) for each condition is shown. The average speed (μm/min), life time (s) and displacement between two consecutives events (μm) are shown. σ is the standard deviation.

We then analyzed the duration of microtubule growth by measuring the growth life time parameter (Fig 2B). Nucleolin and B23 silenced cells showed a shorter growth life time highly significantly different from control cells (P<0.05 and P<0.005 respectively). Conversely, in nucleolin over-expressing cells, a highly significant increase in growth life time was measured compared to control cells (P<0.005). Therefore, our observations highlight a correlation between nucleolin expression level and microtubule growth life time.

To determine whether these changes in microtubule growth speed and life time resulted in shorter or longer microtubules, their polymerization length was analyzed (Fig 2C). For every condition, except B23 over-expressing cells, no significant changes were observed concerning microtubule polymerization length. Therefore, even if the kinetics of growing microtubules was found to be different, it did not result in significant changes in microtubule polymerization length.

To visualize EB3 data, kymographs corresponding to individual comets with long lifetime (>15s) and long polymerization length (>8 μm) are shown for each condition (Fig 2D). The slope of each line corresponds to microtubule growth speed.

In summary, our results show that, on average, a microtubule grows slower and lasts longer in a nucleolin over-expressing cell, compared to a microtubule in a control cell (blue on Fig 2E). Conversely, a microtubule grows during a shorter time in a nucleolin silenced cells (red on Fig 2E). For B23, the more pronounced traits are observed for silenced cells, where on average a microtubule grows faster during a shorter period of time (red on Fig 2F).

**Fig 3:**
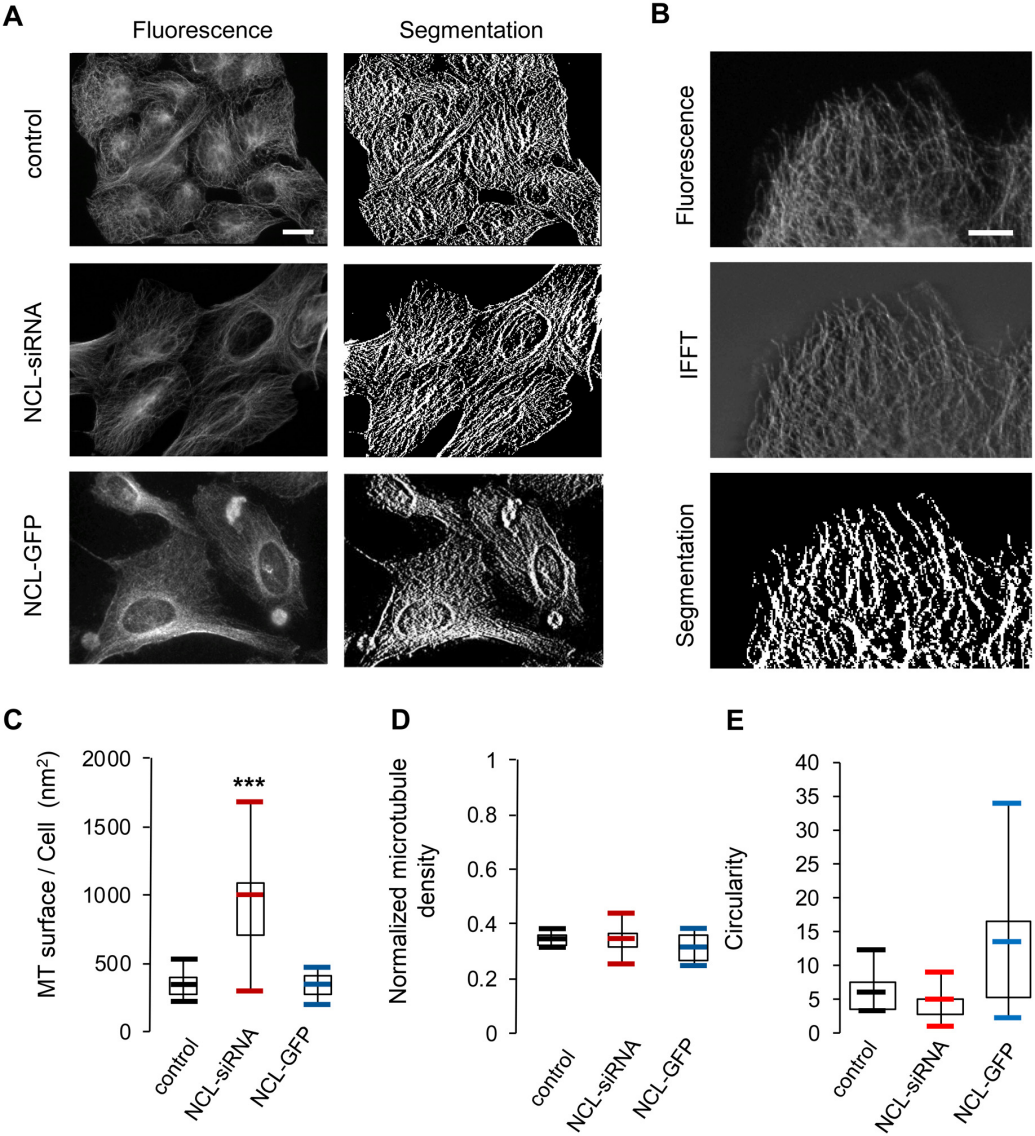
Nucleolin expression does not influence microtubules density. U2OS cells were either transfected with NCL-siRNA or NCL-GFP plasmid and after 4 days were fixed for tubulin immunofluorescence detection (A). The first column shows the immunofluorescence picture of the best focus image among the z-stack. The second column shows the segmentation picture. (B) Details of one control cell showing the different steps of the analysis IFFT: Inverse Fast Fourier Transform. Scale bars represent = 10μm. (C) Quantification of the microtubule surface per cell for control, NCL-siRNA and NCL-GFP cells. The asterisks indicate that the mean value differs from the control condition with the Wilcoxon-Mann-Whitney two-sample rank test at 0,5% (*** P<0.005). Cells were individually analyzed (control: 7 cells; NCL-siRNA: 21 cells; NCL-GFP: 8 cells). (D) Quantification of the microtubule density obtained by dividing the microtubule surface by the whole cell surface for control (7), NCL-siRNA (21) and NCL-GFP (8) cells. (E) Quantification of cell circularity for control (35 cells); Nucleolin-siRNA (32 cells) and NCL-GFP (45 cells). (** P<0.05)

To determine whether the observed differences in microtubule dynamics were associated with a global modification of microtubule density, analysis of immuno-labeled tubulin images was performed in nucleolin depleted or over-expressing cells (Fig 3A and 3B). The surface labeled by the microtubule network was found to be significantly higher (P< 0.005) in nucleolin silenced cells compared to control (Fig 3C). However when these values were normalized by the cell surface to calculate microtubule density, no more differences could be detected between nucleolin silenced cells and control (Fig 3D). This effect is due to the larger size of nucleolin silenced cells, that we previously reported [19]. Therefore, differences in microtubule dynamics observed in nucleolin silenced or over-expressing cells do not correlate with a variation in microtubule density. Altogether, our data highlight that nucleolin expression level, like B23, plays a role on the dynamics of growing microtubules, without affecting global microtubule density.

As microtubules can be involved in cell shape, we determined if changes in nucleolin expression could affect cell morphology. For this, a circularity index of the cells in the different conditions was calculated (Fig 3E). Circularity is define as the ratio of the square of the perimeter to (4*π*Area). A value of 1 would correspond to a perfectly round cell, while higher value correspond to shape that deviate from a circle. Control cells (35 cells) and nucleolin depleted cells (32 cells) have an average circularity index of 5.9 and 4.6 respectively. By contrast NCL-GFP (45 cells) strongly deviate from a round cell with an average circularity of 13.2. More experiments would be required to determine if this change in cell shape is a direct or indirect consequence of the effect of nucleolin on microtubule dynamic.

### Nucleolin expression affects the repartition of all microtubule subpopulations

**Fig 4:**
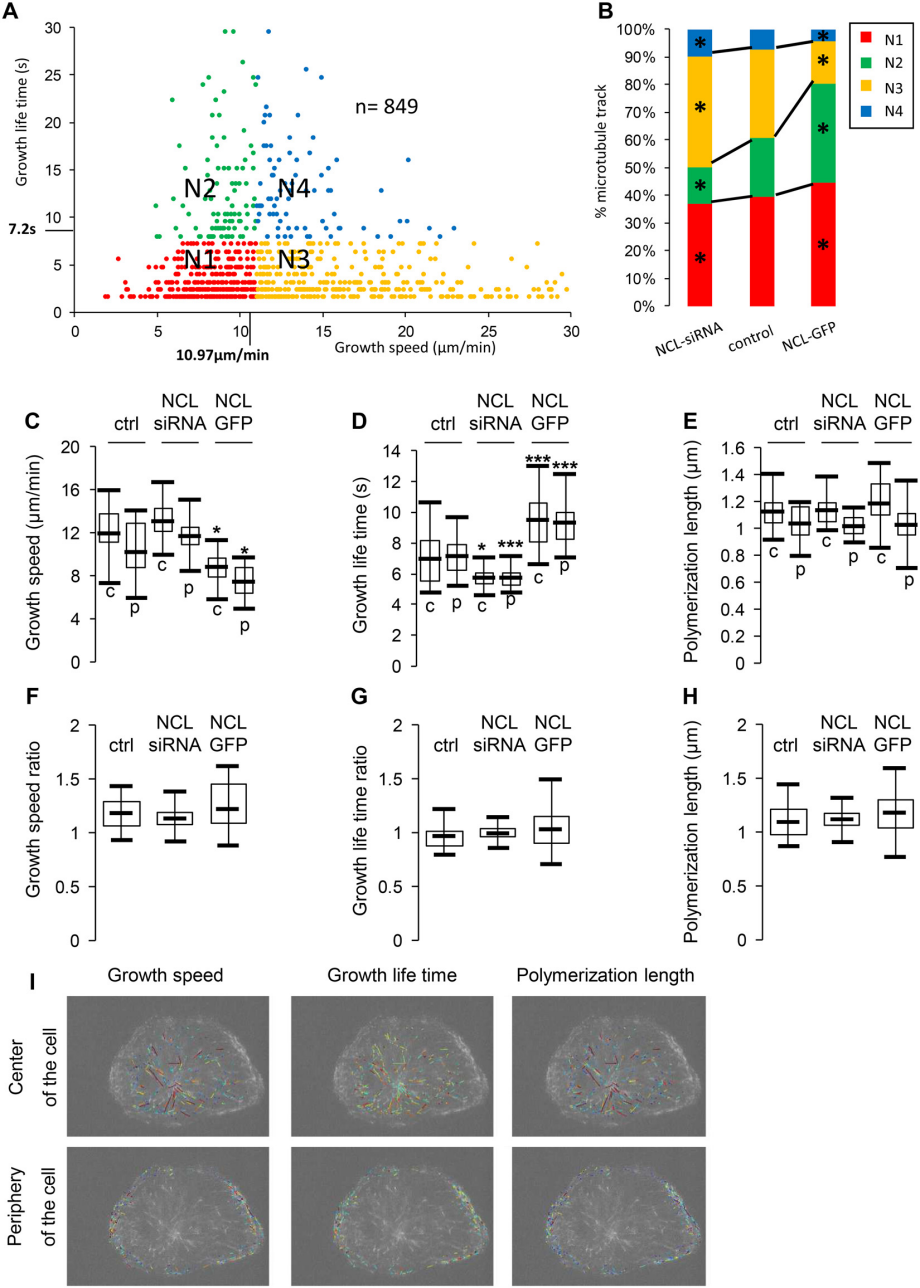
Nucleolin expression affects all microtubule subpopulations. (A) Quadrant scatter plot showing the classification of microtubule growth tracks according to their growth speed (x-axis) and life time (y-axis). Example of microtubule growth tracks (849 tracks) detected in a single control cell. Microtubule growth tracks are represented by the filled circles. They are classified in four categories: N1 “slow and short lived” [red], N2 “slow and long lived” [green], N3 “fast and short lived” [yellow] and N4 “fast and long lived” [blue]. The fast and slow groups as well as the short-lived versus long-lived groups were spilt using the mean growth parameters of control cells [speed: 10.97μm/min and life time: 7.2s]. (B) Effect of nucleolin on the proportion of microtubules growth tracks in each subpopulation depicted in A. For each condition, the number of microtubules in each subpopulation was added for all the cells and the percentage was then calculated. The same color code serves to represent the four populations as in (A). The asterisks indicate a different repartition from the control condition (* P<0.05; χ² test). (C)-(E): Box and whisker plots represent tracks for growth speed (C), growth life time (D) and polymerization length (E) in the center and periphery of the cells (“c” and “p” respectively). For each condition (control, nucleolin depletion and nucleolin over expression), cells were individually analyzed (control: 18 cells; NCL-siRNA: 24 cells; NCL-GFP: 16 cells), in two ROI corresponding to the center and the periphery of the cells. The mean value of the parameters was individually calculated in each cell. For each condition, the mean value of the parameters was obtained by averaging the value calculated for each cell. On the graphs, boxes indicate 25 and 75% quartiles and the whiskers extend to 1.5 times of the interquartile range. The asterisks indicate that the mean values differ from the control condition (in the corresponding region: center vs periphery of the cell) with the Wilcoxon-Mann-Whitney two-sample rank test at different significance levels: 5% (* P<0.05), 1% (*** P<0.01). (F)-(H): Box and whisker plots represent the ratio for growth speed (F), growth life time (G) and polymerization length (F) of the center over the periphery of the cell. The ratio of the parameters was individually calculated in each cell. For each condition, the mean value of the parameters was obtained by averaging the value calculated for each cell. On the graphs, boxes indicate 25 and 75% quartiles and the whiskers extend to 1.5 times of the interquartile range. (I): Microtubule growth tracks are color coded by speed (left panel), life time (middle panel) or polymerization length (right panel) in the center (upper panels) or in the periphery (lower panels) of the cell displayed in Fig 1A.

Microtubule dynamic properties differ according to microtubule subcellular localization; the central region of the cells being dominated by fast moving microtubules. In order to decipher the effect of nucleolin on the different microtubule categories, microtubule growth tracks were classified on the basis of two dynamics parameters: growth speed and life time (Fig 4). Four classes of microtubules were defined as “slow and short lived” [red], “slow and long lived” [green], “fast and short lived” [yellow] and “fast and long lived” [blue] (Fig 4A) according to previous work [27]. The fast and slow groups as well as the short-lived versus long-lived were spilt using the mean growth parameters of control cells [speed: 10.97μm/min and life time: 7.2s] (see Table 1). The repartition of growth tracks in the four classes was calculated in whole individual cells under- or over-expressing nucleolin vs control cells, and presented as a color-coded bar graphs for easier comparison. Nucleolin expression levels affect the repartition of microtubules in the four different categories (Chi2 test 0.001) (Fig 4B). In nucleolin depleted cells, the proportion of “slow” microtubules decreases (red and green categories) (Fig 4B). On the opposite, in nucleolin over-expressing cells, the percentage of “fast” microtubules increases (yellow and blue categories) (Fig 4B). To further determine if the effect of nucleolin on microtubule dynamics is dependent on the localization of the microtubules in the cells, the different parameters were calculated for peripheral or central microtubule populations (Fig 4I) in each cell population (control, nucleolin siRNA and NCL-GFP) (Fig 4C–4I). As expected, central (c) microtubules show a higher growth speed compared to peripheral (p) microtubules in all cell categories (Fig 4C and 4F). As described in Fig 2B, the microtubules growth life time was significantly reduced upon nucleolin depletion and increased in cells overexpressing nucleolin for both central and peripheral microtubules (Fig 4D) while no significant changes were observed for the polymerization length (Fig 4E). Thus, these different measurements show that nucleolin affects the repartition of all microtubules arguing for a global role of nucleolin in the control of microtubule dynamics, independently of their subcellular localization.

### Effect of nucleolin on microtubule pauses

**Fig 5:**
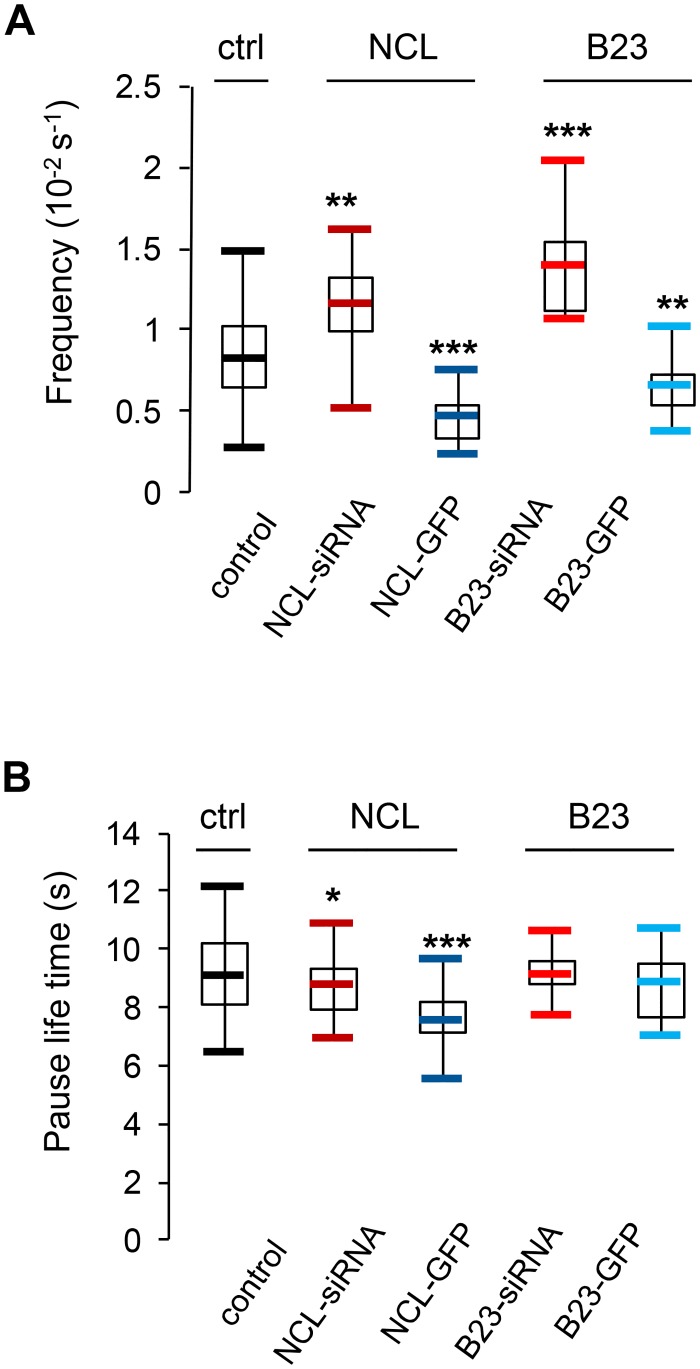
Nucleolin expression affects microtubule pauses. Analysis of pause events in control [black], nucleolin depleted [dark red], nucleolin over expressing [dark blue], B23 depleted [light red] and B23 over expressing cells [light blue]. The asterisks indicate that the mean value differs from the control condition with the Wilcoxon-Mann-Whitney two-sample rank test at different significance levels: 10% (* P<0.1), 5% (** P<0.05), 0,5% (*** P<0.005). (A) Pause frequency (10^-2^s^-1^) is calculated by dividing the number of pause events by the time microtubules spend in growth. Cells were individually analyzed (control: 18 cells; NCL-siRNA: 24 cells; NCL-GFP: 15 cells; B23-siRNA: 11 cells; B23-GFP: 15 cells). (B) Pause life time (s) represents the mean time between the disappearance and the recovering of EB3-TagRFP signal during pause events.

We next wanted to take advantage of the ability to follow microtubule dynamics, to estimate microtubule pause and catastrophe parameters, in the same cells. Since the EB3 signal temporarily disappears between two polymerization phases without apparent growth or depolymerization (Fig 1B, see pause), it is only an indirect marker of pause events. However, by linking two spatially and temporally adjacent growth subtracks, it is possible to deduce the frequency of pause events as well as the pause life time, as previously reported using the same cell line [27, 28]. Thus, we decided to analyze whether nucleolin or B23 expression levels modify microtubule pause frequency and life time (Fig 5 and Table 2).

**Table 2:** Pause and frequency parameters.

	Pauses		Catastrophes			
	frequency (10^−2^ s^-1^)	life time (s)	frequency (10^−3^ s^-1^)	speed (μm/min)	life time (s)	displacement (μm)
control	0.83 (σ = 0.59)	9.14 (σ = 1.45)	1.41 (σ = 1.18)	17.77 (σ = 4.85)	2.57 (σ = 0.63)	0.73 (σ = 0.27)
NCL siRNA	1.15 (σ = 0.46)	8.71 (σ = 1.08)	1.27 (σ = 0.79)	23.33 (σ = 4.70)	2.81 (σ = 0.53)	1.07 (σ = 0.26)
NCL-GFP	0.46 (σ = 0.65)	7.52 (σ = 1.03)	0.39 (σ = 0.87)	14.56 (σ = 5.04)	2.24 (σ = 0.66)	0.53 (σ = 0.24)
B23 siRNA	1.38 (σ = 0.54)	9.10 (σ = 1.08)	1.69 (σ = 0.99)	31.47 (σ = 5.06)	3.11 (σ = 0.47)	1.62 (σ = 0.42)
B23-GFP	0.65 (σ = 0.61)	8.81 (σ = 1.20)	1.00 (σ = 0.84)	17.15 (σ = 8.45)	2.44 (σ = 0.60)	0.67 (σ = 0.37)

The frequency (10^-2^s^-1^ or 10^-3^s^-1^) of pause and catastrophe are shown. The mean values of pause life time (s) and the catastrophe parameters are shown [speed (μm/min) / life time (s) / displacement (μm)]. σ is the standard deviation.

The frequency of pauses was significantly higher in nucleolin and B23 depleted cells (P<0.05 and P<0.005 respectively), whereas in cells over expressing nucleolin or B23, pause frequency was significantly lower (P<0.005 and P<0.05 respectively) compared to control cells (Fig 5A). Pause life time was then calculated by measuring the time between the disappearance of the EB3-tagRFP signal and the regain of growth (Fig 5B). In addition to being less frequent in nucleolin over-expressing cells, pause life time was also significantly shorter (P<0.005, Fig 5B). The reverse was also true for nucleolin silenced cells (P<0.1, Fig 5B). Altogether, our results show that nucleolin and B23 expression levels significantly modulate microtubule pause frequency and duration for nucleolin level.

### Nucleolin inhibits catastrophes

**Fig 6:**
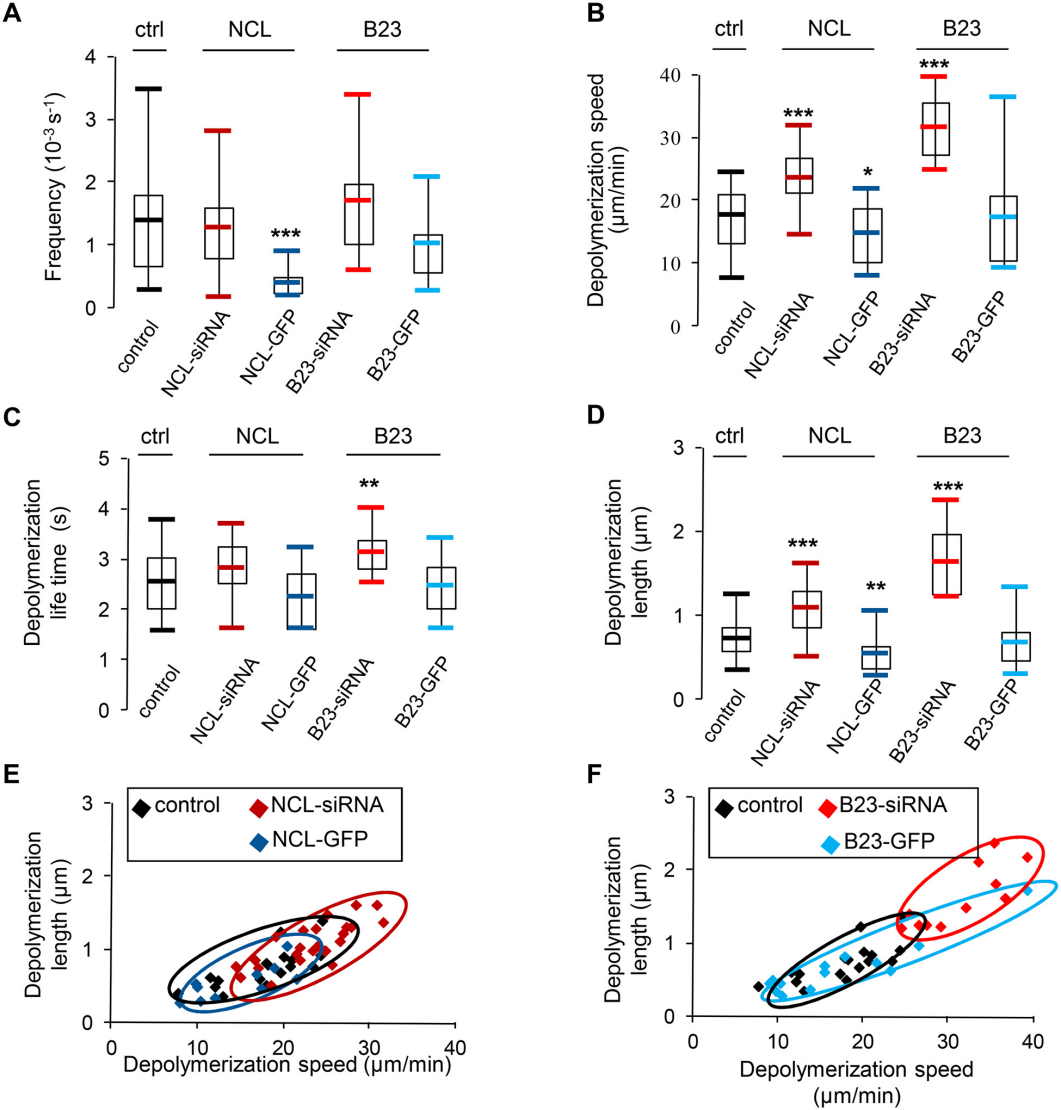
Nucleolin expression affects microtubule catastrophes. Analysis of catastrophe events in control [black], nucleolin depleted [dark red], nucleolin over expressing [dark blue], B23 depleted [light red] and B23 over expressing cells [light blue]. The asterisks indicate that the mean value differs from the control condition with the Wilcoxon-Mann-Whitney two-sample rank test at different significance levels: 10% (* P<0.1), 5% (** P<0.05), 0,5% (*** P<0.005). (A) Catastrophe frequency (10^-3^s^-1^) is calculated by dividing the time microtubules spend in growth (B)-(D) Catastrophe parameters are deduced by linking two temporally adjacent growths spatially spaced out by a Bgap (Backward Gap). The mean values of speed (B), life time (C) and length (D) during catastrophes were measured in each cell and reported on the graphs. (E) and (F): 2D graphs showing the repartition of the cells according to depolymerization speed (x-axis) and length (y-axis). Control [black], nucleolin over expressing [dark blue] and nucleolin depleted [dark red] (E) or B23 over expressing [light blue] and B23 depleted [light red] (F) cells are represented by the squares. (A-D) Cells were individually analyzed (control: 17 cells; NCL-siRNA: 24 cells; NCL-GFP: 10 cells; B23-siRNA: 11 cells; B23-GFP: 14 cells)

We then analyzed microtubule parameters during catastrophe events (Fig 6 and Table 2) which are characterized by a temporarily disappearance of the EB3-tagRFP signal and a reappearance (see Fig 1B, catastrophe). The PlusTipTracker software detects catastrophe events by linking temporally close growths, spatially spaced out by a Backward Gap (Bgap). Like pause frequency, catastrophe frequency was significantly lower in nucleolin over expressing cells (P<0.005, Fig 6A). In addition to being less frequent, catastrophes in nucleolin over expressing cells were also found with a slower depolymerization speed (P<0.1, Fig 6B) and a comparable life time (Fig 6C), thus resulting in a shorter depolymerization length (P<0.05, Fig 6D). Conversely, in nucleolin depleted cells a faster depolymerization speed was observed compared to control cells (P<0.05, Fig 6B) and a comparable life time (Fig 6C), thus resulting in a longer depolymerization length (P<0.05, Fig 6D). The same was also true in B23 depleted cells (Fig 6B–6D). In summary, our results show that on average a microtubule depolymerizes faster and on a longer distance in nucleolin silenced cells compared to control cells (red on Fig 6E), while microtubule tend to depolymerize slower on a shorter length in nucleolin overexpressing cells (blue on Fig 6E). For B23, the more pronounced traits are also observed for silenced cells (red on Fig 6F), with the same trends than for nucleolin.

In conclusion, catastrophe events are more frequent when cells over-express nucleolin and depolymerization speed is anti-correlated to the amount of nucleolin level, as for polymerization events. However, for catastrophe events, it is the depolymerization life time which tends to be constant rather than the life time as for growth events. Therefore, microtubule depolymerization length is, like speed, anti-correlated to nucleolin level, suggesting a role for nucleolin (and B23) in microtubule stabilization.

### Nucleolin is required for the stabilization of acetylated microtubule during cold induced depolymerization

**Fig 7:**
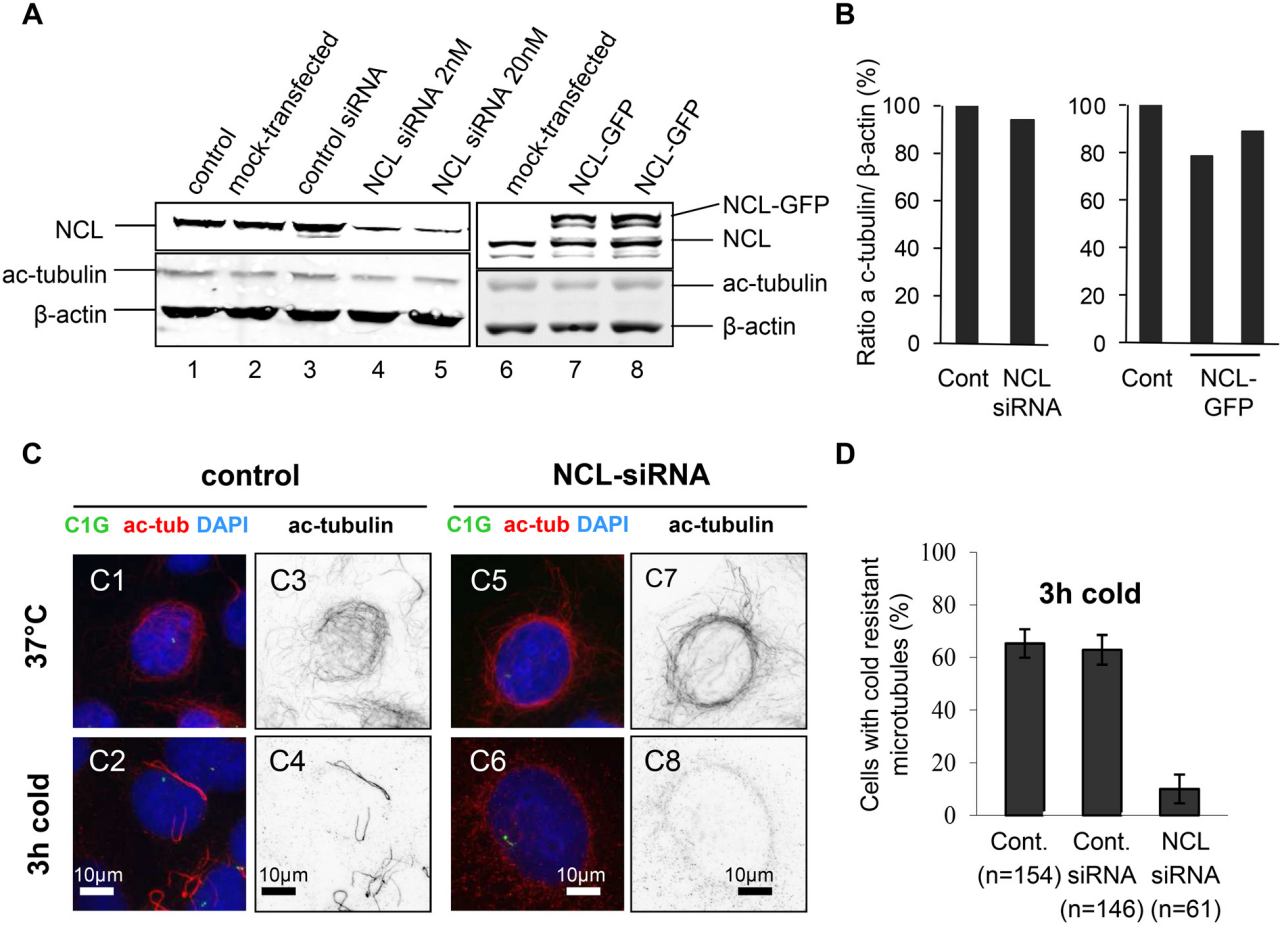
Loss of cold-resistant acetylated microtubules in nucleolin silenced cells. (A) Inhibition of nucleolin expression by siRNA. Western-blot analysis of whole cell extracts from untransfected control, mock transfected, control siRNA and nucleolin siRNA (siRNA concentration 2nM or 20nM) transfected cells. Cells were harvested four days after transfection and protein extracts were analyzed by western-blot using an anti- nucleolin polyclonal antibody (secondary antibody coupled to IRdye800) and acetylated tubulin (ac-tubulin) and β-actin monoclonal antibodies (secondary antibody coupled to Alexa680). (B) Quantifications of the fluorescent western blots, displayed in (A), expressed as a ratio of ac-tubulin over β-actin. The normalized expression level of acetylated tubulin was set to 100% in control cells (NCL siRNA 94.22% / NCL-GFP: 78.75% and 89.32%). (C) Microtubule regrowth after cold induced depolymerization in untransfected control and nucleolin siRNA transfected U2OS-centrin-1-GFP cells. Co-visualization of acetylated tubulin (ac-tub), centrin-1-GFP (C1G) and nuclear counterstain (DAPI) shown as 3-color merged image and black and white images of acetylated tubulin (inverted dynamics), before depolymerization first row), and immediately after depolymerization (second row). Centrin-1-GFP detection was enhanced with a GFP booster [green], acetylated tubulin was detected with a monoclonal antibody (secondary antibody coupled to Alexa555) [red on the 3-color merged images and black on black and white images]. Scale bars represent 10μm. (D) Quantification of the percentage of cells incubated in cold for 3h (C2, C6) exhibiting microtubules resistant to depolymerization. These cell classes are displayed for untransfected control cells (Cont.), control siRNA transfected cells (Cont. siRNA) and nucleolin siRNA transfected cells (NCL-siRNA). The values are expressed as percentages of the total number n of the cells studied. Error bars represent the standard deviation from two independent experiments (standard deviation of Bernoulli experiments = $\sqrt{p\left(1-p\right)/n}$).

To get further insights into this new function of nucleolin on microtubule dynamics, we investigated the effects of nucleolin depletion on stabilized microtubules that are characterized by the presence of acetylated tubulin. We used U2OS cells stably expressing centrin-1-GFP in which acetylated tubulin was detected (Fig 7). The inhibition or over-expression of nucleolin had no effect on the accumulation of ac-tubulin (Fig 7A and 7B) and on the networks of stabilized microtubules (Fig 7C and 7D). When cells were incubated at 4°C for three hours most microtubules were depolymerized as expected, and few microtubules, resistant to depolymerization were observed in 60% of control cells (Fig 7C2–7C4 and 7D, Cont. n = 154). These stable microtubules were clustered and not oriented toward the centrosome identified by the centrin-1-GFP signal (Fig 7C2). Similar results were observed in control siRNA transfected cells (Fig 7D, Cont. siRNA, n = 146). Interestingly these resistant microtubules were almost not observed in nucleolin depleted cells (Fig 7C6–7C8 and 7D). Indeed, only 10% of nucleolin depleted cells retained these stabilized microtubules after cold incubation (Fig 7D, NCL siRNA, n = 61). This observation is in agreement with a destabilization of microtubules linked to nucleolin depletion.

## Discussion

In this report, we have characterized the effects of nucleolin expression on microtubule dynamics by showing that nucleolin level is anti-correlated to microtubule growth speed, like that of the previously shown *in vitro* microtubule stabilizer B23 [6]. Using the PlusTipTracker software package [25, 26], we have studied global microtubule dynamic parameters in live cells by detecting and analyzing microtubule plus-ends with the EB3-tagRFP binding protein. Our results show that, without affecting cellular density of microtubules (Fig 3), nucleolin over-expressing cells exhibit a significantly slower speed for polymerizing microtubules (Fig 2A), while nucleolin silenced cells exhibit a significantly faster speed for depolymerizing microtubules (Fig 6B), in agreement with a new role of nucleolin in microtubule stabilization. The same trend was measured in cells overexpressing or silenced for B23, another microtubule stabilizer protein. In polymerizing microtubules, growth speed variations are found to be anti-correlated with growth life time variations (Fig 2B), while in depolymerizing microtubules, growth speed variations are found to be correlated with depolymerization length variations (Fig 6D). Nucleolin over expression is also associated with a lower frequency of pause events (Fig 5) and of microtubule catastrophes (Fig 6A). This stabilization effect of nucleolin on microtubule was confirmed in a cold induced microtubule depolymerization experiment, in which nucleolin depletion was associated with a more efficient depolymerisation of microtubules (Fig 7). Altogether, our results demonstrate a new cytoplasmic role for nucleolin in microtubule stabilization. These observations could be the consequences of several mechanisms involving nucleolin.

**Fig 8:**
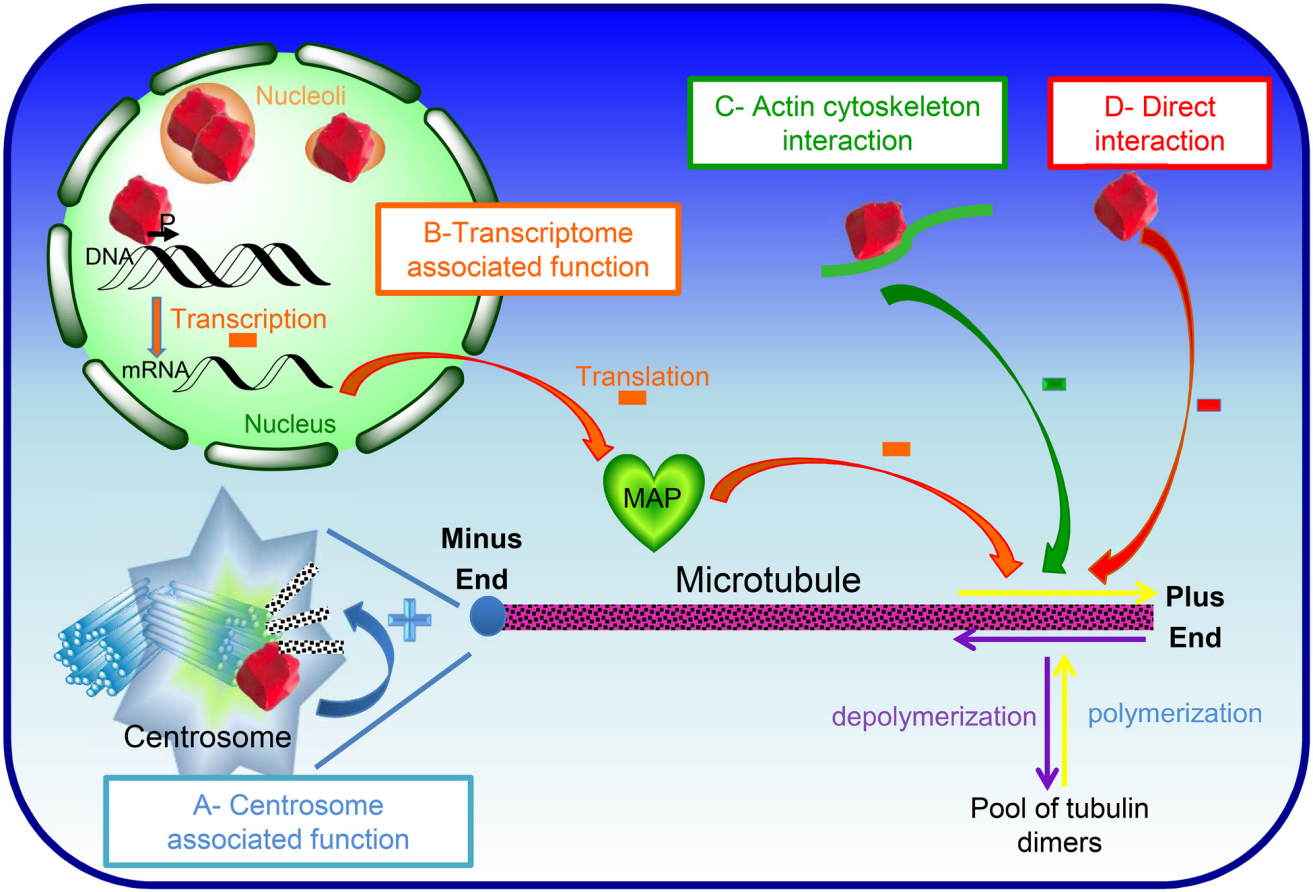
Nucleolin can affect microtubules stability through multiple pathways. Based on previous studies and our unpublished observations, four hypotheses can be drawn to explain nucleolin’s role on microtubule dynamics, simplified in the current scheme by polymerization and depolymerization arrows (respectively in purple and yellow) at the Minus and plus Ends. The first hypothesis derives from nucleolin’s role on centrosome function (A-centrosome associated function, in blue). Indeed, nucleolin (in red) localizes at the mature centriole, interacting with ninein and γTuRC, where it is involved in microtubule nucleation and anchoring [15]. The second hypothesis is based on the control of RNAP II gene transcription (B- transcriptome associated function, in orange), which is regulated by nucleolin binding to a specific gene regulating element (our unpublished results). The third hypothesis is through binding to microtubule associated proteins or with actin cytoskeleton, known to interact with microtubule (C-Actin cytoskeleton interaction, in green). The fourth hypothesis is that nucleolin could directly interact with microtubules (D- Direct interaction, in red), thereby resulting in slower and longer-lasting polymerizing microtubules.

The first possibility (Fig 8A) is that changes in microtubules dynamics in nucleolin depleted or over-expressing cells is a consequence of nucleolin’s role on centrosomal microtubule nucleation and anchoring through its interaction with ninein and γ-tubulin [15]. This hypothesis does not explain how nucleolin could affect microtubules plus ends when localized on microtubules minus ends. The second possibility (Fig 8B) is that nucleolin acts indirectly on microtubule dynamics by down-regulating the expression of a microtubule associated Protein (MAP, green heart on Fig 8). Indeed, transcriptomic analysis of cells depleted in nucleolin shows that the expression of several genes linked to microtubules are deregulated (P. Bouvet, unpublished data). It is therefore possible to explain a reduction on microtubule polymerization growth if nucleolin down-regulates the expression of a MAP stabilizing microtubules. The third possibility (Fig 8C) is that nucleolin acts indirectly on microtubule dynamics by interacting with the actin cytoskeleton. Indeed, several studies have functionally linked cytoplasmic nucleolin to actin cytoskeleton [30, 31]. Given the strong interconnection between microtubules and actin filaments, it will be interesting to further study if the expression level of nucleolin affects microtubules dynamics through other cytoplasmic interacting partners like actin. The fourth and last possibility (Fig 8D) is that nucleolin acts on microtubule dynamics by binding directly to microtubule plus ends. Indeed, a proteomic study has recently identified nucleolin as a microtubule associated protein [20] suggesting that nucleolin could affect microtubule dynamics by directly interacting with them. However, despite several attemps to co-localize nucleolin along the microtubules in vivo using immunofluorescence techniques, we were not able to confirm that nucleolin is directly associated with microtubules in the cells (data not shown). Future work should discriminate between these different hypotheses.

Altogether, our studies show that nucleolin has two major effects on microtubule regulation: nucleolin participates in the control of microtubule nucleation and anchoring at the centrosome [17] and nucleolin expression level modulates microtubule polymerization in the cytoplasm (this study).

Our observations are in agreement with a similar role of nucleolin and B23 on microtubule dynamics. Nucleolin and B23 are two structurally different proteins that have been shown to interact with each other [32] and numerous functions in common have already been reported. Both proteins are involved in ribosome biogenesis within the nucleoli and in the same manner as nucleolin, B23 is localized at the centrosome during interphase [33] and its absence leads to microtubule polymerization defects [16]. However, the function and localization of nucleolin at centrosome are independent of B23 [15]. Interestingly, B23 has been shown to stabilize microtubules by antagonizing the Eg5-mediated microtubule destabilizing function [6]. As nucleolin interacts with hundreds of proteins within the cell, we cannot exclude that the effects of nucleolin that we observed might involve indirect mechanisms via its interaction in the cytoplasm with key microtubule regulators such as Eg5. Thus, given that the effects of nucleolin on microtubule dynamics are similar to that of B23, it is tempting to hypothesize that the microtubule related functions of nucleolin might implicate Eg5 and/or B23. To this end, it will be interesting to decipher the interactome of nucleolin in the cytoplasm.

In interphase cells, regulation of microtubule dynamics is essential for numerous processes including cell architecture [34], intracellular trafficking and cell adhesion [35, 36], cell polarization [37] and migration [38]. Interestingly, nucleolin has been associated to microtubule dependent intracellular trafficking [13, 39] and has been implicated in cell adhesion and migration [9, 12, 40] It would be interesting to reevaluate the function of nucleolin in these processes by taking into account the involvement of nucleolin microtubule stabilization described herein. Indeed, in this report we show that nucleolin expression levels affect the different subpopulations of microtubules at the center and at the periphery of the cells (Fig 4), arguing for a global role of nucleolin on microtubule dynamics independently of their subcellular localization. Thus, nucleolin controls microtubules dynamics in the center of the cell as well as in the cell periphery where microtubules are involved in cell migration and adhesion.

In addition, during mitosis, the fine regulation of microtubule dynamics is also critical for proper chromosome attachment and segregation. Disturbed microtubule dynamics leads to chromosome missegregation and aneuploidy [41]. Interestingly, nucleolin absence results in defects in kinetochore-microtubule attachments, chromosome missegregation and aneuploidy [19, 42, 43] that can partially be explained by the presence of extra centrosomes in nucleolin depleted cells, that lead to the formation of multipolar spindles when cells reach mitosis [15, 19, 43]. Furthermore, the effect of nucleolin on microtubule dynamics described in this report may exist in mitosis; indeed, a role of nucleolin on mitotic chromosome segregation [44] by controlling microtubule dynamics, is possible and needs to be studied.

Microtubules also play important role in cancer related cellular processes. As nucleolin is deregulated in many cancer cells [8, 44, 45], its over expression might have critical consequences on microtubule dynamics, thus favoring the initiation and/or progression of cancer. Deciphering the role of nucleolin protein level deregulation on microtubule dynamics is a new approach to better understand the multiple contribution of nucleolin in cell transformation and tumorigenesis.
